# The Role of KRAS Mutations in Cortical Malformation and Epilepsy Surgery: A Novel Report of Nevus Sebaceous Syndrome and Review of the Literature

**DOI:** 10.3390/brainsci11060793

**Published:** 2021-06-16

**Authors:** Chiara Pepi, Luca de Palma, Marina Trivisano, Nicola Pietrafusa, Francesca Romana Lepri, Andrea Diociaiuti, Francesca Diomedi Camassei, Giusy Carfi-Pavia, Alessandro De Benedictis, Camilla Rossi-Espagnet, Federico Vigevano, Carlo Efisio Marras, Antonio Novelli, Ingmar Bluemcke, Nicola Specchio

**Affiliations:** 1 Rare and Complex Epilepsy Unit, Department of Neuroscience, Bambino Gesù Children’s Hospital IRCCS, Member of European Reference Network EpiCARE, 00165 Rome, Italy; chiara.pepi@opbg.net (C.P.); luca.depalma@opbg.net (L.d.P.); marina.trivisano@opbg.net (M.T.); nicola1.pietrafusa@opbg.net (N.P.); giusy.carfipavia@opbg.net (G.C.-P.); 2 Translational Cytogenomics Research Unit, Laboratory of Medical Genetics, Bambino Gesù Children Hospital, IRCCS, 00165 Rome, Italy; francescaromana.lepri@opbg.net (F.R.L.); antonio.novelli@opbg.net (A.N.); 3 Genodermatosis Unit, Genetics and Rare Diseases Research Division, Bambino Gesù Children’s Hospital, IRCCS, Member of European Reference Network SKIN, 00165 Rome, Italy; andrea.diociaiuti@opbg.net; 4 Department of Laboratories—Pathology Unit, Bambino Gesù Children’s Hospital, IRCCS, 00165 Rome, Italy; francesca.diomedi@opbg.net; 5 Neurosurgery Unit, Department of Neurosciences, Bambino Gesù Children’s Hospital, IRCCS, Member of European Reference Network EpiCARE, 00165 Rome, Italy; alessandro.debenedictis@opbg.net (A.D.B.); carloefisio.marras@opbg.net (C.E.M.); 6 Neuroradiology Unit, Imaging Department, Bambino Gesù Children’s Hospital, IRCCS, 00165 Rome, Italy; mcamilla.rossi@opbg.net; 7 Neuroradiology Unit, NESMOS Department, Sapienza University, 00165 Rome, Italy; 8 Department of Neuroscience, Bambino Gesù Children’s Hospital IRCCS, Member of European Reference Network EpiCARE, 00165 Rome, Italy; federico.vigevano@opbg.net; 9 Institute of Neuropathology, Member of European Reference Network EpiCARE, University Hospitals Erlangen, 91054 Erlangen, Germany; Ingmar.Bluemcke@uk-erlangen.de

**Keywords:** KRAS genetic variants, nevus sebaceous syndrome, pediatric epilepsy surgery, hippocampal sclerosis, focal cortical dysplasia, RAS pathway

## Abstract

The rare nevus sebaceous (NS) syndrome (NSS) includes cortical malformations and drug-resistant epilepsy. Somatic RAS-pathway genetic variants are pathogenetic in NS, but not yet described within the brain of patients with NSS. We report on a 5-year-old boy with mild psychomotor delay. A brown-yellow linear skin lesion suggestive of NS in the left temporo-occipital area was evident at birth. Epileptic spasms presented at aged six months. EEG showed continuous left temporo-occipital epileptiform abnormalities. Brain MRI revealed a similarly located diffuse cortical malformation with temporal pole volume reduction and a small hippocampus. We performed a left temporo-occipital resection with histopathological diagnosis of focal cortical dysplasia type Ia in the occipital region and hippocampal sclerosis type 1. Three years after surgery, he is seizure-and drug-free (Engel class Ia) and showed cognitive improvement. Genetic examination of brain and skin specimens revealed the c.35G > T (p.Gly12Val) KRAS somatic missense mutation. Literature review suggests epilepsy surgery in patients with NSS is highly efficacious, with 73% probability of seizure freedom. The few histological analyses reported evidenced disorganized cortex, occasionally with cytomegalic neurons. This is the first reported association of a KRAS genetic variant with cortical malformations associated with epilepsy, and suggests a possible genetic substrate for hippocampal sclerosis.

## 1. Introduction

Nevus sebaceous syndrome (NSS; also known as Schimmelpenning–Feuerstein–Mims syndrome) is a rare neurocutaneous disorder in which nevus sebaceous (NS) is found in association with congenital abnormalities of the brain, eye, and/or skeleton [1]. Usually on the scalp or face, NS is an epidermal nevus originating from the sebaceous glands and characterized by a waxy, yellow-orange patch of skin.

A previous report focused on the neurological phenotype found no clear distinction between the different types of epidermal nevi [1], though linear epidermal keratinocytic nevi and organoid sebaceous nevi often appear similar. Nevertheless, distinction between the two nevi does indicate different genetic substrates: somatic de novo HRAS, KRAS, and NRAS genetic variants occurring during embryogenesis having been found to be pathogenic in NS, whereas keratinocytic nevi may carry genetic variants beyond the RAS pathway, such as FGFR3 and PIK3CA [2,3,4].

The phenotypic expression is pleomorphic, ranging from solely cutaneous involvement to severe epilepsy due to hemispheric malformation in association with alteration in bone size [2,5,6]. Severity is usually associated with the percentage of mosaicism [7]. Generally, the earlier the mutation occurs, the more widespread is the cutaneous, brain, and skeletal involvement. In up to 25% of cases, increased cellular proliferation and neoplastic transformation occurs, presumably as a result of HRAS and KRAS gene variants causing aberrant activation of Raf–MEK–ERK and phosphoinositol-3-kinase signaling [5].

Neurological involvement in NSS is evident in 5–15% of patients [8,9], most frequently being different degrees of intellectual disability, epileptic seizures (especially epileptic spasms), and hemiparesis. Epilepsy occurs in anywhere from 38% to 96% of patients with NSS and neurological involvement [10,11,12]. Notably, onset of epilepsy is usually earlier than in other neurocutaneous syndromes, occurring mainly in the first month of life and not later than the first eight months of life [7].

The most common brain malformation is unilateral hemimegalencephaly (HME), occurring in up to 72% of cases [13], and typically ipsilateral to the skin lesion. Multi-lobar alterations of cortical migration and organization might occur. Histological data varies widely (see literature review) but have not included any report of HRAS or KRAS mutations in the brain tissue.

Up to now, KRAS somatic mutations have been described within cerebral specimens, only in arteriovenous malformation (AVM), without a clear genotype–phenotype correlation detected [14,15].

Here we report the first evidence of c.35G > T KRAS genetic variant found in both the skin lesion and a brain specimen in a patient with focal cortical dysplasia (FCD) type Ia, hippocampal sclerosis (HS), and NSS. As the syndrome is extremely rare, we performed a literature review on surgically treated cases with NSS and drug-resistant epilepsy to better delineate the association of NSS with a specific cortical malformation.

## 2. Case Report

**Figure 1:**
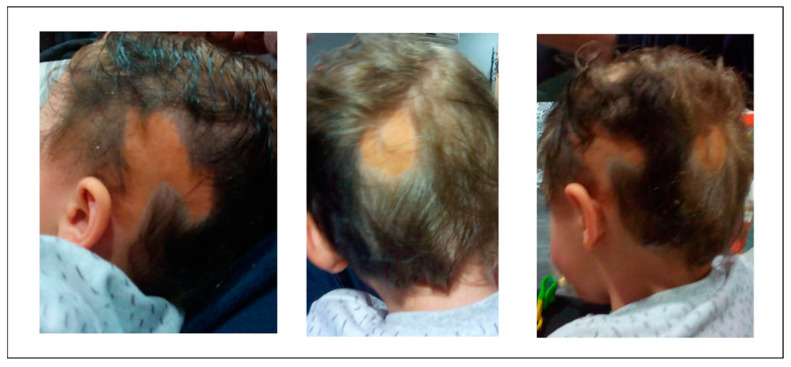
Linear alopecic sebaceous skin lesion in the left temporo-occipital area.

This is a 5-year-old boy who had been delivered at term following an uneventful pregnancy with parents unrelated to each other. No family history of congenital neurocutaneous conditions was reported. At birth, the boy presented with a flat, hairless, yellow-brown linear skin lesion in the left temporo-occipital area, suggestive of nevus sebaceous (Figure 1). Neurological examination revealed asymmetry of spontaneous movements, with preferential use of the left arm and right neglect. Mild motor delay became evident during follow-up. After the first year of age, he also presented language delay, oppositional behavior, and poor social interaction. The Griffith developmental scale at the age of 14 months recorded a total developmental score of 60.

**Figure 2:**
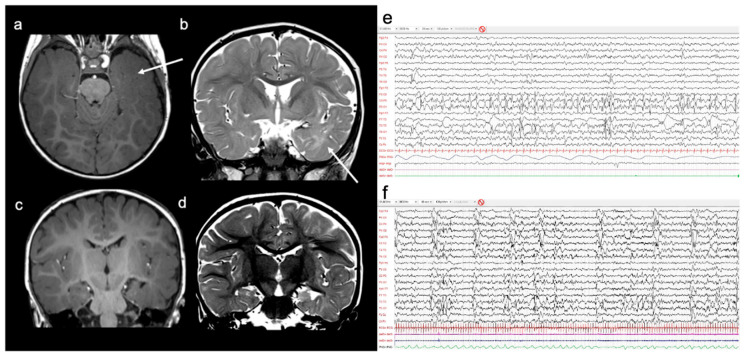
Magnetic resonance imaging T1-weighted images demonstrating left temporal pole volumetric reduction associated with blurring of the cortico-subcortical region (**a**, arrow) and hyperintensity of the white matter on T2-weighted sequences (**b**, arrow). There is incomplete hippocampal inversion on the left (**c**) associated with increased T2 signal (**d**). (**e**) EEG during Wakefulness shows continuous left temporo-occipital slow and epileptiform abnormalities, with sporadic contralateral expression. (**f**) Periodic complexes of slow waves after awakening associated with subtle eyes elevation, recorded in 60 s.

**Figure 3:**
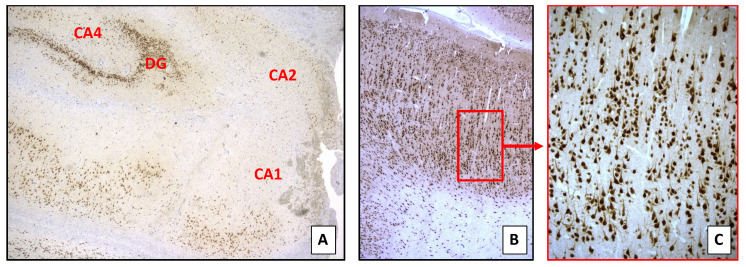
Histopathologic findings. (**A**) Hippocampal sclerosis type 1: pronounced neuronal cell loss along the whole Ammon’s horn (CA); preserved neuronal cellularity of the dentate gyrus (DG). (Neu-N immunostain, 2.5×). (**B**) Occipital lobe focal cortical dysplasia type I: mildly hypercellular cortex harboring small-diameter neurons arranged in distinct microcolumns. Note the cortex–white matter blurring. (Neu-N immunostain, 2.5×). (**C**) Occipital lobe focal cortical dysplasia type I: higher magnification of red insert in (**B**), highlighting the cortex microcolumnar arrangement. (Neu-N immunostain, 20×).

Epilepsy started at 6 months of age, characterized by clusters of epileptic spasms with sudden extensions of upper limbs and trunk lasting 2–3 min. Electroencephalogram (EEG) recordings showed continuous left temporo-occipital epileptiform abnormalities. Brain magnetic resonance imaging (MRI) showed a diffuse left temporal blurring, volume reduction of the temporal pole, and a small hippocampus, with clear T2 hyperintensity (Figure 2a–d). He tried valproic acid and vigabatrin, with initial seizure-free period, until seizures re-appeared after 3 months, characterized by subtle episodes of brief left-ocular deviation and partial loss of awareness. Interictal EEG was characterized by continuous slow waves associated with spikes and poly-spike complexes in the left temporo-occipital region, with frequent and bilateral diffusion, especially during sleep. The ictal video-EEG evidenced periodic diffuse slow waves with superimposed fast activity over the left temporo-occipital region lasting 20–30 s, and was associated with subtle ocular elevation (Figure 2e,f). At the age of 2 years, the patient underwent a left temporo-occipital resection. Histopathological examination documented an FCD type Ia in the occipital region and HS type 1 with preserved cortical organization in the temporal lobe (Figure 3).

During 3 years of postsurgical follow-up, he has remained seizure- and drug-free (Engel class Ia). Cognitive evaluation performed at 4 years of age by Wechsler Preschool and Primary Scale of Intelligence—III reported a total intelligence quotient (TIQ) score of 80 (Verbal IQ: 90, Performance IQ: 73). Postsurgical EEG recordings show an asymmetrical electrical activity without epileptiform abnormalities.

**Figure 4:**
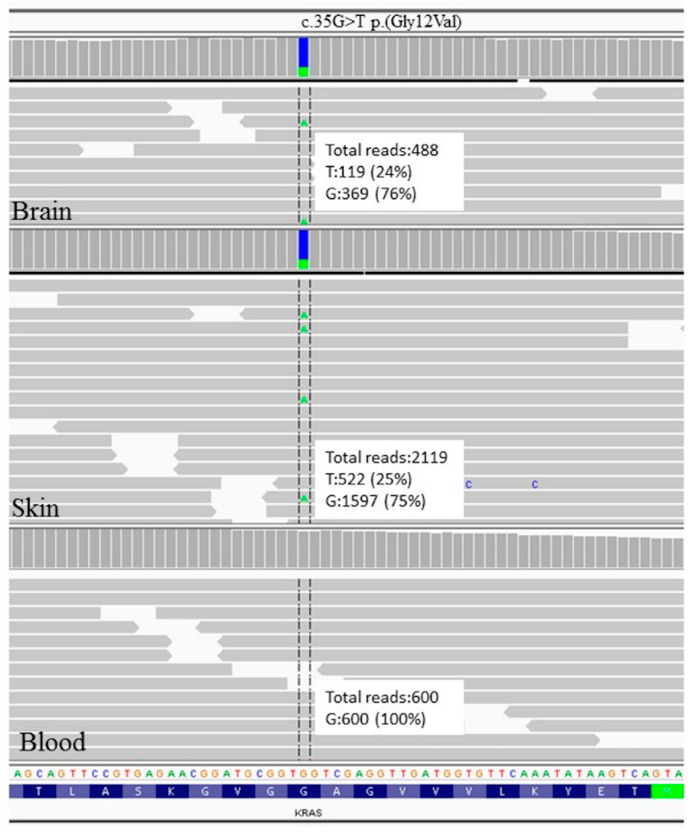
Next-generation sequencing data from brain, skin, and blood samples showing the presence of the c.35G > T (p.Gly12Val) KRAS mutation with a ~25% mosaicism rate both in brain and skin tissue, but absence of the variation in the blood sample, thereby demonstrating the somatic origin of the mutation.

Mutational analysis of HRAS, NRAS, and KRAS genes performed by a custom next-generation sequencing (NGS) panel on DNA extracted from specimens from brain, nevus sebaceous, and blood revealed in the KRAS gene the somatic mutation c.35G > T (p.Gly12Val), already reported and associated with a similar clinical picture, with a ~25% mosaicism rate both in brain- and skin-tissue-derived DNA, but absent in blood-derived DNA (Figure 4).

## 3. Literature Review

We searched PubMed (up to January 2021) for reports on postsurgical seizure outcome in NSS patients who underwent surgery for refractory epilepsy. Search terms “nevus sebaceous surgery hemimegalencephaly” (“nevus, sebaceous of jadassohn”(MeSH Terms) OR (“nevus”(All Fields) AND “sebaceous”(All Fields) AND “jadassohn”(All Fields) OR “sebaceous of jadassohn nevus”(All Fields) OR (“nevus”(All Fields) AND “sebaceous”(All Fields) OR “nevus sebaceous”(All Fields) AND (“surgery”(MeSH Subheading) OR “surgery”(All Fields) OR “surgical procedures, operative”(MeSH Terms) OR (“surgical”(All Fields) AND “procedures”(All Fields) AND “operative”(All Fields) OR “operative surgical procedures”(All Fields) OR “general surgery”(MeSH Terms) OR (“general”(All Fields) AND “surgery”(All Fields) OR “general surgery”(All Fields) OR “surgery s”(All Fields) OR “surgerys”(All Fields) OR “surgeries”(All Fields) AND (“hemimegalencephaly”(MeSH Terms) OR “hemimegalencephaly”(All Fields) OR “hemimegalencephalies”(All Fields) collectively returned 27 articles. Our initial screen of studies included those that:

1. Reported single-patient data concerning postsurgical outcome, MRI features, and surgical procedures of NSS patients who underwent surgery for epilepsy;
2. Clearly reported the presence of NS; and
3. Were written in English.

Nine publications (including reviews, case reports, comments, and editorials) satisfied criterion 1 and were included for full-text review for relevance; one case report was excluded because the type of epidermal nevus was not clearly stated. All were written in English. Eleven cases from eight publications were reviewed, and relevant clinical, imaging, and histological data are summarized in Table 1. One patient was reported consecutively in two papers [16,17] (marked in Table 1). All of the studies reviewed precede the consensus classification of the ILAE Task Force of Focal Cortical Dysplasia [18].

Data on seizure semiology and histological findings are sparse, and NSS patients are mainly included in hemispherectomy case series [16,17,19]. Seizure semiology was reported in six patients: one presented epileptic spasms, two had focal myoclonic/clonic seizures, and the remainder each had either focal tonic seizures, focal seizure with a clear bilateral diffusion, or motor features of seizure were not reported. Malformations of cortical development were the most common neuroradiological findings, involving the whole hemisphere in six patients (55%), two or three lobes (temporal, parietal, or occipital) in four patients (36%), while one patient (9%) had only a single lobe (temporal) malformation. Description of histological findings from brain specimens was reported in three patients, each describing a disorganized cortical architectural pattern with two also reporting a clear cytomegaly with giant dysmorphic neurons.

Regarding the surgical procedures, these subtotaled as:

- Five (45%) anatomical and functional hemispherectomies;
- Three (27%) temporo-parieto-occipital multilobar resections;
- Three (27%) temporal lobectomies/lesionectomies.

Follow-up was reported in ten cases (91%). After a median follow-up of three years, eight patients (73%) were in Engel class I and three patients (27%) had seizure persistence.

## 4. Discussion

We report the c.35G > T KRAS somatic mutation in a patient with NSS and a complex brain malformation characterized by HS and FCD type I in the occipital lobe. The mutation was detected in both skin and brain specimens, but not in a blood sample. This is the first documented KRAS mutation in a brain specimen of cortical malformation related to epilepsy.

### 4.1. Clinical Features

#### Neurologic Features

Our patient displayed the classical “neurological variant” of NSS [1], consisting of multilobar or hemispheric cortical malformation, global neurocognitive delay, and epileptic seizures (especially epileptic spasms). He presented with an early onset epileptic encephalopathy, promptly treated with epilepsy surgery. He experienced a major improvement in developmental level in the postoperative follow-up, whereas seizure persistence in such cases is supposed to be a major aggravating factor for intellectual disability [24,25].

### 4.2. Extra-Neurologic Features

#### Skin Anomalies

Epidermal genetic mosaicism in patients with NSS might present as epidermal nevi, which may be subdivided into keratinocytic epidermal nevi and organoid nevi, the latter including NS and follicular nevus. NS is a congenital hamartoma that may contain any components of the skin. It was the first described type of epidermal nevus, and likely represents the most common variety [11]. Cutaneous distribution of NS usually follows the linear patterns known as “lines of Blaschko” [7]. In infancy and childhood (“first stage”, before puberty), due to the quiescence of sebaceous glands, NS typically appears as a yellow, flat patch; when it occurs on the scalp, it appears as a yellowish, hairless area. At puberty, NS often thickens and develops a velvety and verrucous surface, due to an increased number of mature sebaceous glands (“second stage”). In the third stage, benign and malign transformation might occur.

### 4.3. Other Manifestations

Eye abnormalities: Ocular and palpebral abnormalities are observed in nearly 40% of patients [26]. The most frequent abnormalities detected are lipodermoids and coloboma, followed by corneal opacities, retinal changes (including scarring, degeneration, and detachment), ptosis, macrophthalmia, and conjunctival growths. Loss of vision in the involved eye is common. There is an association between optic nerve hypoplasia and NS in some cases [27].

Skeletal anomalies: Skeletal anomalies were found in 50% of 74 patients with NS reviewed by Grebe [28]. Most of these anomalies are ipsilateral to the cutaneous NS. Skeletal manifestations of NS syndrome have also been reported [29], including bone hyper-or hypoplasia, kyphoscoliosis, and skull/craniofacial asymmetry.

Cardiovascular anomalies: patent ductus arteriosus, coarctation of the aorta, and septal defects in NSS patients may be seen [30].

Other organs abnormalities: Less commonly, NSS is associated with anomalies involving other organs, such as the genito-urinary system [28] and endocrine system [31,32,33,34].

### 4.4. Neuroradiological Features

The prominent neuroradiological feature from previous reports of patients with NSS and drug-resistant epilepsy is HME, which is characterized by an alteration in both neuronal migration and proliferation [35]. Milder malformations, like patchy macrogyria and microgyria, are more rarely reported [36].

In our patient, brain enlargement was not clearly evidenced from the brain MRI; rather, the MRI demonstrated a volumetric reduction of the temporal pole associated with diffuse cortico-subcortical blurring of the left temporo-occipital region and incomplete hippocampal inversion with increased T2 signal of the hippocampus. This radiological feature is peculiar, considering that the most typical radiological characteristics are unilateral HME, with ventricular enlargement, and white matter hyperintensity [36]. Periventricular heterotopia has also been reported in NSS [36].

Other described brain MRI findings, such as vascular malformations, agenesis of the corpus callosum, Dandy–Walker syndrome, myelomeningocele, Arnold–Chiari malformation, and tumors (astrocytoma, pineal germinoma, choroid plexus papilloma, and lipoma of the corpus callosum) were also not evident with our patient [1,37,38,39].

### 4.5. Epilepsy Surgery

Based on anatomo-electro-clinical correlations [40], we performed a left temporo-occipital resection resulting in an excellent seizure outcome (Engel class Ia). Antiseizure medications were tapered off. Cognitive outcome was scored as almost normal at the most recent follow-up [41,42,43].

Epilepsy surgery remains the most appropriate therapeutic perspective in NSS patients. We confirm the literature trend reporting a good post-surgical outcome in 73% of NSS cases. The most common surgery has been hemispherotomy (45%) followed by multilobar resection (27%). A comprehensive evaluation of patients with drug-resistant epilepsy should be done in due time, in order to reach an early diagnosis which can increase the success rate after surgery [44]. In patients with FCDs and different degree of HME, outside NSS, the overall postoperative outcome is 67% after 5 years of follow up [45]. Additionally, looking at series of patients who underwent hemispheric surgery due to HME or wide FCDs, the postoperative outcome is reported to be 67.5% [46].

It has been suggested that an improvement in seizure frequency should be achieved when the cortical resection is sufficiently extensive to include all of the abnormal cortex in patients with NSS [20].

Failures could be explained by the existence of more extensive pathology in the remaining hemisphere than was not delineated on preoperative neuroimaging [41,42,43]. Post-mortem evidence of cortical dysplasia in the non-HME hemisphere has been reported [43].

There are other functional neuroimaging techniques, such as positron emission tomography (PET) and single-photon emission computed tomography (SPECT), that might help to identify epileptogenic foci and possibly allow a more tailored resection [21].

### 4.6. Histopathology

In our patient, the occipital cortical specimen showed a vertical dyslamination with focal micro-columnar organization in terms of a FCD Type I. Such alterations were not detected within the temporal lobe, where there was a blurring of the grey–white matter boundaries with heterotopic neurons within the white matter. In addition, the hippocampal tissue showed segmental cell loss within the sectors of the pyramidal cell layer, a granule cell dispersion and granule cell loss, consistent with HS Type 1.

The striking feature is the coexistence of FCD type I in the occipital lobe, quite far away from the temporal lobe where we found only some heterotopic neurons in the white matter and an HS type 1.

The histological diagnosis from cerebral specimens in NSS was rarely reported in past published cases, thereby excluding clear definition of FCD type I and FCD type II [13,22,23].

The most common cerebral histopathological findings in NSS patients are descriptive report of a severe cortical dysplasia and loss of the normal six-layer architecture of the cortex, including focal loss of obvious lamination and haphazard orientation of neurons within the cortex. Increased number of neuronal cells was observed in the deep white matter, constituting neuronal heterotopia.

Most of the reports concluded that the findings were concordant with the diagnosis of HME [47].

### 4.7. Genetic Features

Mosaicism is a basic feature of nevus sebaceous syndrome that was inferred clinically by several authors [48,49]. The discovery that a subgroup of keratinocytic epidermal nevi is caused by oncogenic FGFR3 and PIK3CA mutations with mosaicism [50] was a revelation of these nevi and multiorgan involvement because FGFR and PIK3CA participate in embryogenesis, angiogenesis, and tissue homeostasis. This discovery was followed by the identification of FGFR3 mutation in keratinocytic epidermal nevus syndrome in a female with epilepsy since infancy [51]. Almost simultaneously, it was demonstrated that nevus sebaceous and linear sebaceous nevus syndrome are also caused by postzygotic HRAS and KRAS mutations [2,52,53].

The KRAS gene is located on chromosome 12p12.1 and consists of six exons. Germline mutations in KRAS have been identified in patients with cardio-facio-cutaneous (CFC) syndrome, Noonan syndrome [54,55], and Costello syndrome [56]. Somatic KRAS mutations frequently occur in lung, colorectal, and pancreatic cancers [57,58]. The somatic KRAS c.35G > A mutation is the most frequent KRAS mutation in human cancers according to the COSMIC database. However, widespread expression of KRAS c.35G > A is not tolerated during mouse embryonic development [59].

The first KRAS G12D mutation was reported in an infant with epidermal nevus, polycystic kidneys, and rhabdomyosarcoma [60]. This mosaic KRAS G12D mutation was detected in both the epidermal component of the nevus and in the rhabdomyosarcoma, but not in the unaffected tissues or normal skin or blood. This observation strongly suggests a somatic mosaicism. Since the first case, Levinsohn and other authors have identified four additional nevus sebaceous patients with a somatic p.Gly12Asp KRAS mutation [52,61]. These four patients only have nevus sebaceous, with no other characteristics reported.

Up to 95% of NS have heterozygous mutations in HRAS, with the mutation c.37G > C (p.Gly13Arg) accounting for the majority [2,52,53]. Most other NS have mutations in KRAS (c.35G > A or c.35G > T) and, unlike keratinocytic epidermal naevi, no mutations have been found in NRAS, FGFR3, or PIK3CA. Tissue microdissection showed that the HRAS/KRAS mutations occurred in NS keratinocytes and not in adjacent non-lesional skin, regional fibroblasts, or peripheral-blood-derived genomic DNA, thus confirming the postzygotic mosaicism basis of NS. Around 10% of NS had two mutations (second mutation in HRAS or additional mutation in BRAF), but these were not associated with distinct clinical or histological features, suggesting possible redundancy with respect to MAPK or PI3K pathway activation [2,62]. The identification of mutations in HRAS and NRAS in NS provides insight into the pathophysiology of this hamartoma, defining NS as a mosaic RASopathy. Moreover, the aberrant activation of Raf–MEK–ERK and PI3K signaling provides an explanation for the cellular proliferation and increased susceptibility to tumors.

In previous reports, no genetic analyses were performed within brain specimens.

Up to now, brain KRAS somatic mutations were described only in AVM. Nikolaev et al. [63] first reported KRAS mutations in intracranial AVM samples. In 45/72 (63%) of their intracranial AVM specimens, they found somatic KRAS mutations that were predicted to activate KRAS-mediated signaling. In subsequent papers, a great variability in mutant prevalence was reported [14,15,64], possibly due to the lack of standardized sampling methods. Currently, there are no obvious genotype–phenotype correlations that can be used to predict whether a somatic mutation will be detected and, if so, which gene will be mutated (i.e., KRAS, BRAF).

Within the malformation of cortical development (MCD), it is known that there is a strong correlation with mTOR pathway deregulation [65].

Over the past decade, increasing evidence suggests that constitutive activation of the mTOR signaling cascade is considered to be a common feature in a subset of MCD. These malformations share some histopathologic and clinical characteristics [66,67], ranging from FCD to HME and megalencephaly. A shared mechanism of pathogenesis is suggested, as the same morphological alterations are seen in all of these pathologies. These common features reflect a disturbance of cellular lineage and cellular growth [68,69].

Therefore, the term “mTORopathies” (mTOR-pathway-related malformations) has been introduced to define a spectrum of MCDs characterized by altered cortical architecture, abnormal neuronal/glial morphology, and intractable seizures as a consequence of a deregulation of the mTOR signaling.

FCD could potentially represent the result of somatic gene mutations within the mTOR pathway. Indeed, several FCD cohort studies have shown the proportion of cells showing mutant allele in the resected brain tissue to vary from 1% to 14% [70,71]. In comparison, HME specimens from HME patients, in whom a more extensive area of the brain is abnormal, the mutant allelic proportions tend to be higher, ranging between 2.5% and 44% [72,73,74]. Hypothetically, these findings could be suggestive of a “gene dosage effect” in which greater mutant allele proportion is associated with more extensive and often more severe cortical malformations [74].

HME is caused by variants of genes related to mTOR pathways and their activators (AKT3, PIK3CA, RHEB). This malformation is characterized by a higher variant allele frequency than FCD type II, with variants detected mainly in dysmorphic neurons and balloon cells [75]. Our case did not show this hallmark of HME but was characterized by a KRAS variant in the brain and skin that was absent in the peripheral blood. This KRAS variant has previously been reported as causative of NS [2,52,76].

KRAS is involved in the MAPK pathways and could be related to cortical malformation because RAS/extracellular-signal-regulated kinase signaling pathways are potential activators of mTORC1 and mTORC2 upstream [77]. Recently, the possible association between somatic RAS-pathway variants and FCD type II has been highlighted [78].

FCD type I, as found in our patient, had not previously been linked to RAS or mTOR pathway mutations in brain specimens. We believe our case report is the first to report a RAS mutation to be associated with FCD type I, though the nature of this association has yet to be established. The *SLC35A2* gene is the only genetic variant previously found within a FCD type I specimen, and this variant is not known to be involved in mTOR or RAS regulation. The *SLC35A2* gene encodes a UDP-galactose transporter, a member of the nucleotide-sugar transporter family that transports galactose from the cytosol or nucleus into Golgi vesicles; hence, its mutation is involved in congenital disorders of glycosylation [79].

Our patient also evidenced an HS. In a publication reporting a patient with mesio-temporal drug-resistant epilepsy and a KRAS germline mutation, association with the hippocampus was hypothesized as through ERK activation, which is crucial for homeostasis and particularly responsible for both the long-term potentiation of synaptic transmission and the protein-kinase modulation of dendritic K^+^ channels [80].

The major strength of our report is the long-standing seizure freedom following the removal of malformed tissue, even after complete withdrawal from antiseizure medication. That the KRAS mutation was present only in the removed pathological brain and skin tissue, and not in the blood, is concordant with the hypothesis that this mutation might be linked to the epilepsy in this case.

## 5. Conclusions

This case demonstrated that mosaic KRAS genetic variants can be associated with cortical malformation in the context of NSS. KRAS mutations could lead to epileptogenicity by either altering mTOR activation through MAPK pathways or altering synaptic transmission in hippocampal regions. Further studies are needed to understand how the same mutation could lead to different cortical malformation in the same patient. We confirm that epilepsy surgery in patients with drug-resistant epilepsy is efficacious and may facilitate cognitive development.

## Figures and Tables

**Table 1:** Summary of relevant clinical, imaging, and histological data from individual cases in previously published reports.

Report	MRI	Surgery	Engel Class I	Seizure Semiology	Histology	Follow-Up
[13]	L HME	L hemispherectomy	Yes	Focal motor	Disorganized cortical architectural pattern and neuronal cytomegaly	1 y
[16] *	HME	Hemispherectomy	Improvement	n.a.	n.a.	n.a.
[17]	R HME	R hemispherectomy	Yes	n.a.	n.a.	3 y
[17]	R HME	R hemispherectomy	No (II)	n.a.	n.a.	3 y
[17] *	R HME	R hemispherectomy	Yes	n.a.	n.a.	3 y
[19]	R HME	R temporal lobectomy	No (II)	Focal myoclonic/clonic	n.a.	4 y
[20]	R pariet, occip, temp	R multilobar lobectomy	Yes	Focal to bilateral	n.a.	11 m
[20]	L pariet, occip, temp	L multilobar lobectomy	Yes	Focal myoclonic/clonic	n.a.	11 m
[20]	L HME	L hemispherectomy	Yes	Focal tonic	n.a.	4.6 y
[21]	L temp, occip	L temporal, occipital lobectomy	Yes	n.a.	Disorganized cortical architectural pattern, neuronal and glial heterotopias	7 m
[22]	R temp	R temporal lobectomy	No (IV)	Epileptic spasms	n.a.	n.a.
[23]	R pariet, temp	R temporal lesionectomy	Yes	n.a.	Disorganized cortical architectural pattern, neuronal and glial heterotopias, giant neurons	6 y

MRI: magnetic resonance imaging; HME: hemimegaloencephaly; L: left; R: right; temp: temporal; occip: occipital; pariet: parietal; n.a.: not available. * same patient.

## Data Availability

Data are available at investigator site.

## Abbreviations

EEG, electroencephalogram; FCD, focal cortical dysplasia; HME, hemimegalencephaly; MRI, magnetic resonance imaging; NGS, next-generation sequencing; NSS, nevus sebaceous; NSS, nevus sebaceous syndrome; PIQ, performance intelligence quotient; TIQ, total intelligence quotient; VIQ, verbal intelligence quotient.
